# Pan-Cancer Analysis of Genomic and Prognostic Characteristics Associated With Coronavirus Disease 2019 Regulators

**DOI:** 10.3389/fmed.2021.662460

**Published:** 2021-08-11

**Authors:** Jian Zhang, Huali Jiang, Kunpeng Du, Tao Xie, Baiyao Wang, Chengcong Chen, Bohong Cen, Yawei Yuan, Jiacai Ye

**Affiliations:** ^1^Department of Radiation Oncology, Affiliated Cancer Hospital and Institute of Guangzhou Medical University, State Key Laboratory of Respiratory Diseases, Guangzhou Institute of Respiratory Disease, Guangzhou, China; ^2^Guangzhou Medical University, Guangzhou, China; ^3^Department of Cardiovascularology, Tungwah Hospital of Sun Yat-sen University, Dongguan, China

**Keywords:** pan-cancer, COVID-19 regulators, genetic alterations, methylation, prognosis

## Abstract

**Background:** Cancer patients are alleged to have poor coronavirus disease 2019 (COVID-19) outcomes. However, no systematic or comprehensive analyses of the role and mechanisms of COVID-19 receptor-related regulators in cancer are available.

**Methods:** We comprehensively evaluated the genomic alterations and their clinical relevance of six COVID-19 receptor-related regulators [transmembrane serine protease 2 (TMPRSS2), angiotensinogen (AGT), angiotensin-converting enzyme 1 (ACE1), solute carrier family 6 member 19 (SLC6A19), angiotensin-converting enzyme 2 (ACE2), and angiotensin II receptor type 2 (AGTR2)] across a broad spectrum of solid tumors. RNA-seq data, single nucleotide variation data, copy number variation data, methylation data, and miRNA–mRNA interaction network data from The Cancer Genome Atlas (TCGA) of 33 solid tumors were analyzed. We assessed the sensitivities of drugs targeting COVID-19 receptor-related regulators, using information from the Cancer Therapeutics Response Portal database.

**Results:** We found that there are widespread genetic alterations of COVID-19 regulators and that their expression levels were significantly correlated with the activity of cancer hallmark-related pathways. Moreover, COVID-19 receptor-related regulators may be used as prognostic biomarkers. By mining the genomics of drug sensitivities in cancer databases, we discovered a number of potential drugs that may target COVID-19 receptor-related regulators.

**Conclusion:** This study revealed the genomic alterations and clinical characteristics of COVID-19 receptor-related regulators across 33 cancers, which may clarify the potential mechanism between COVID-19 receptor-related regulators and tumorigenesis and provide a novel approach for cancer treatments.

## Introduction

The severe acute respiratory syndrome coronavirus 2 (SARS-CoV-2) virus has resulted in the ongoing coronavirus disease 2019 (COVID-19) pandemic. As of July 7, 2021, there are 184,820,132 confirmed cases and 4,002,209 deaths, with the numbers still surging worldwide (1). With the continued increase in cases and affected regions, patients with chronic conditions, such as cancer, have been disproportionately affected (2–5). The COVID-19 pandemic has been identified as a global health emergency by the World Health Organization (WHO).

Respiratory inflammation is activated by the renin–angiotensin–aldosterone system (RAS), which maintains the blood pressure by angiotensin II (Ang II) and is catalyzed by the angiotensin-converting enzyme (ACE). Angiotensinogen (AGT) is the protein precursor of Ang II (6, 7). Ang II receptor type 2 (AGTR2), a member of the G-protein coupled receptor 1 family, functions as a receptor for Ang II. ACE2 degrades Ang II, counteracting its chronic effects, and serves as the SARS-CoV-2 receptor. ACE2 is also a molecule present on the surface of various cell types, including type II alveolar cells, bronchial transient epithelial secretory cells, endothelial cells, intestinal epithelium cells, and uterine epithelial cells (8). The spike protein (S protein) of SARS-CoV binds to cell surface ACE2 receptors (9). ACE1, homologous to the ACE2 gene, may be involved in the progression of diseases caused by several human coronaviruses (10, 11). Transmembrane serine protease 2 (TMPRSS2), a member of the serine protease family, facilitates human coronavirus infections (SARS-CoV and SARS-CoV-2) *via* proteolytic cleavage of the ACE2 receptor, which promotes viral uptake and cleavage of coronavirus spike glycoproteins, activating glycoproteins for host cell entry (12–14). Solute carrier family 6 member 19 (SLC6A19), a SARS-CoV-2 co-receptor, is a neutral amino acid transporter and forms a heterodimer with ACE2 (15). However, the genomic alterations and prognostic characteristics of COVID-19 receptor-related regulators in cancer are still unclear.

The clinical symptoms of COVID-19 range from asymptomatic to severe cardiopulmonary disease (16–18). Enhanced expression of ACE2 and immunosuppressive states caused by malignancies and anticancer treatments, such as chemotherapy or surgery, contribute to more severe disease in older patients with COVID-19 (19, 20). Recent studies also identified that aberrant expression of ACE2 receptor-related regulators is associated with the activation of several cancer-associated pathways (21–23). Therefore, it is of great clinical significance to clarify the genomic and clinical characteristics of the six ACE2 receptor-related regulators among 33 solid tumors for the management and treatment of tumor patients with COVID-19.

## Methods

### Dataset Acquisition and Preprocessing

The Genotype-Tissue Expression (GTEx) dataset (V7.0) (https://commonfund.nih.gov/GTEx/) was used for gene expression analysis in normal tissues from healthy individuals. The tumor-associated data are composed of mRNA Seq data, clinical data, single nucleotide variation (SNV) data, copy number variation (CNV) data, and methylation data, which were collected from The Cancer Genome Atlas (TCGA) (https://portal.gdc.cancer.gov/). Reverse phase protein array (RPPA) data were obtained from The Cancer Proteome Atlas (TCPA) (https://tcpaportal.org/tcpa/index.html). The Genomics of Drug Sensitivity in Cancer (GDSC) database (www.cancerrxgene.org) was used to investigate the correlation between gene expression and drug sensitivity.

Samples from 33 solid cancer types were investigated in the final analysis, namely, adrenocortical carcinoma (ACC), bladder urothelial carcinoma (BLCA), breast invasive carcinoma (BRCA), cervical squamous cell carcinoma and endocervical adenocarcinoma (CESC), cholangiocarcinoma (CHOL), colon adenocarcinoma (COAD), lymphoid neoplasm diffuse large B-cell lymphoma (DLBC), esophageal carcinoma (ESCA), glioblastoma multiforme (GBM), head and neck squamous cell carcinoma (HNSC), kidney chromophobe (KICH), kidney renal clear cell carcinoma (KIRC), kidney renal papillary cell carcinoma (KIRP), acute myeloid leukemia (LAML), brain low-grade glioma (LGG), liver hepatocellular carcinoma (LIHC), lung adenocarcinoma (LUAD), lung squamous cell carcinoma (LUSC), mesothelioma (MESO), ovarian serous cystadenocarcinoma (OV), pancreatic adenocarcinoma (PAAD), pheochromocytoma and paraganglioma (PCPG), prostate adenocarcinoma (PRAD), rectum adenocarcinoma (READ), sarcoma (SARC), skin cutaneous melanoma (SKCM), stomach adenocarcinoma (STAD), testicular germ cell tumors (TGCT), thyroid carcinoma (THCA), thymoma (THYM), uterine corpus endometrial carcinoma (UCEC), uterine carcinosarcoma (UCS), and uveal melanoma (UVM).

### mRNA Expression Analysis

For mRNA differential expression analysis between paired tumor and normal samples, TCGA mRNA expression was normalized using RNA-Seq by Expectation-Maximization (RSEM). The number of samples for each cancer type ranged from 48 to 1,098, where only 14 cancer types that harbored over 10 pairs of tumor and normal samples were incorporated into analyses, namely, BLCA, BRCA, COAD, ESCA, HNSC, KICH, KIRC, KIRP, LIHC, LUAD, LUSC, PRAD, STAD, and THCA. Gene expression values were represented as RNA-Seq by Expectation-Maximization (RSEM) normalized data (24). The genes with a fold change (FC) <2 and significance false discovery rate (FDR) <0.05 underwent further analysis.

### Subtype Analysis

Expression subtype analysis was used to find clinically relevant genes that may affect cancer subtype. To make the analysis feasible, the number of subgroups in a given subtype was at least 10, leaving 11 cancer types for gene analysis. We analyzed 11 cancer types for ACE2 receptor-relevant genes using a Student's *t*-test (n_subtype = 2) and ANOVA test (n_subtype > 2). The method used for the clinically relevant analysis depends on the number of subgroups in each cancer subtype.

### Survival Analysis

For expression survival analysis, mRNA expression and clinical survival data were merged by the sample barcode. Tumor samples were divided into “high” and “low” gene expression groups using the median RSEM value. The R package “survival” was used to fit the survival time and survival status for the two groups. A Cox Proportional-Hazards model was calculated for each gene using the R package. Genes that had a Kaplan–Meier log-rank test *p*-value <0.05 were retained.

### SNV Analysis

SNV data of 33 cancer types (*N* = 8663) were investigated. SNV oncoplot (or waterfall plot) was generated by maftools (25). The TCGA SNV data includes the following variant type values: Missense_Mutation, Silent, 5' Flank, 3' UTR, RNA, In_Frame_Del, Nonsense_Mutation, Splice_Site, Intron, 5' UTR, In_Frame_Ins, Frame_Shift_Del, Nonstop_Mutation, 3' Flank, Frame_Shift_Ins, and Translation_Start_Site. The Silent, Intron, IGR, 3' UTR, 5' UTR, 3' Flank, and 5' Flank were filtered out for SNV percentage calculation. The percentage of SNVs in each gene's coding region was calculated by the number of mutated samples divided by the number of cancer samples. SNV data and clinical overall survival data were combined, and the R package was used to estimate the survival difference between mutated and non-mutated genes.

### CNV Analysis

CNV raw data from 33 cancer types (*N* = 11,495) were investigated and processed with GISTICS2.0 (26). The CNV was divided into heterozygous and homozygous CNV subtypes, which represented the occurrence of CNV on one chromosome or two chromosomes, respectively. The homozygous or heterozygous CNV profile showed the percentage of homozygous or heterozygous CNV, including CNV amplification and deletion percentages for each gene in each cancer. The percentage of CNV subtypes was calculated using GISTIC-processed CNV data. Only genes with >5% CNV were considered significant. As the method has been employed by Schlattl et al. (27), the mRNA expression and CNV data were merged by a sample's TCGA barcodes. The association between paired mRNA expression and CNV percentage were detected based on a Pearson product–moment correlation coefficient and *t*-distribution.

### Methylation Analysis

Methylation data of paired tumor and normal samples across 14 cancer types (*N* = 10,129) were investigated. The mRNA expression and methylation data were merged by a sample's TCGA barcode. The association between paired mRNA expression and methylation was tested based on a Pearson product–moment correlation coefficient and *t*-distribution. The mRNA expression and methylation data of the regulators were merged *via* the TCGA barcode. The association between paired mRNA expression and methylation data was calculated using the Pearson's product–moment correlation coefficient, followed by a *t*-distribution test. *p*-values were adjusted by the FDR, and genes with an FDR ≤ 0.05 were retained. Further analysis was carried out on genes that were significantly influenced by genome methylation. Methylation data and clinical overall survival data were combined, and the methylation level of a gene was divided into two groups by median methylation. Cox regression was performed to estimate the hazard (risk of death). If the Cox coefficient was <0, the high methylation group showed a poorer survival, the Hyper_worse defined as High risk, otherwise defined as Low risk.

### Pathway Activity Analysis

Following the method used by Ye et al. (28), RPPA data from TCPA were used to calculate a score for 7,876 samples. RPPA data of replicates-based normalization (RBN) were median-centered and normalized by the standard deviation across all samples for each component to obtain the relative protein level. The pathway score is the sum of the relative protein levels of all positive regulatory components minus the sum of the relative protein levels of all negative regulatory components in a given pathway. Gene expression was divided into two groups (upregulation group or downregulation group) by the median expression. The difference in the pathway activity score (PAS) between the two groups was determined. When PAS (gene A, upregulation group) was greater than the PAS (gene A, downregulation group), we considered gene A as having an activating effect on a pathway; otherwise, gene A had an inhibitory effect on a pathway.

### miRNA Regulation Network Analysis

miRNA regulation data (*N* = 9,105) was collected from TCGA across 33 cancer types. miRNA expression and gene expression were merged by TCGA barcode. The association between paired mRNA and miRNA expression was tested based on a Pearson product–moment correlation coefficient and *t*-distribution. The *p*-value was adjusted by the FDR, and genes with an FDR of ≤ 0.05 (*R* <0) were retained. The correlation was calculated for all paired samples. In addition, with consideration to the presence of positive regulators (including transcription factors), an miRNA–gene pair with negative correlation was considered as a potential negative regulation pair. Network was generated by visNetwork R packages.

### Drug Sensitivity Analysis

Following the method used by Rees et al. (29), 481 small molecules from the Cancer Therapeutics Response Portal (CTRP) were collected. To analyze the correlation between gene expression and drug sensitivity, the values from the area under the dose–response curve (AUC) for drug and gene expression profiles for all cancer cell lines were downloaded. The Pearson correlation coefficients of transcription levels and AUCs were normalized using Fisher's *z* transformation. The Pearson correlation coefficients of the transcript levels and AUCs were normalized using Fisher's *z* transformation. A Bonferroni-corrected, two-tailed distribution test, with a family-wise error rate of <0.025 in each tail, was used for the *z*-score calculation. Pearson correlation coefficients of annotated drug–target pairs were compared with the same number of correlation pairs generated by random sampling of the correlations. The gene set drug resistance analysis was performed on IC_50_ drug data.

### Statistical Analysis

Correlations between gene expression were evaluated using the Spearman's correlation test. The prognostic significance of the indexes was estimated using Kaplan–Meier survival curves and compared by a log-rank test. The Cox proportional hazards model was used to calculate the adjusted hazard ratio (AHR). All statistical analyses were performed with SPSS version 23.0 (SPSS Inc, Chicago, IL, USA) and R version 3.4.4 (http://www.r-project.org). *p* <0.05 was considered as statistical significant.

## Results

### mRNA Expression and Subtypes of ACE2 Receptor Regulators

**Figure 1:**
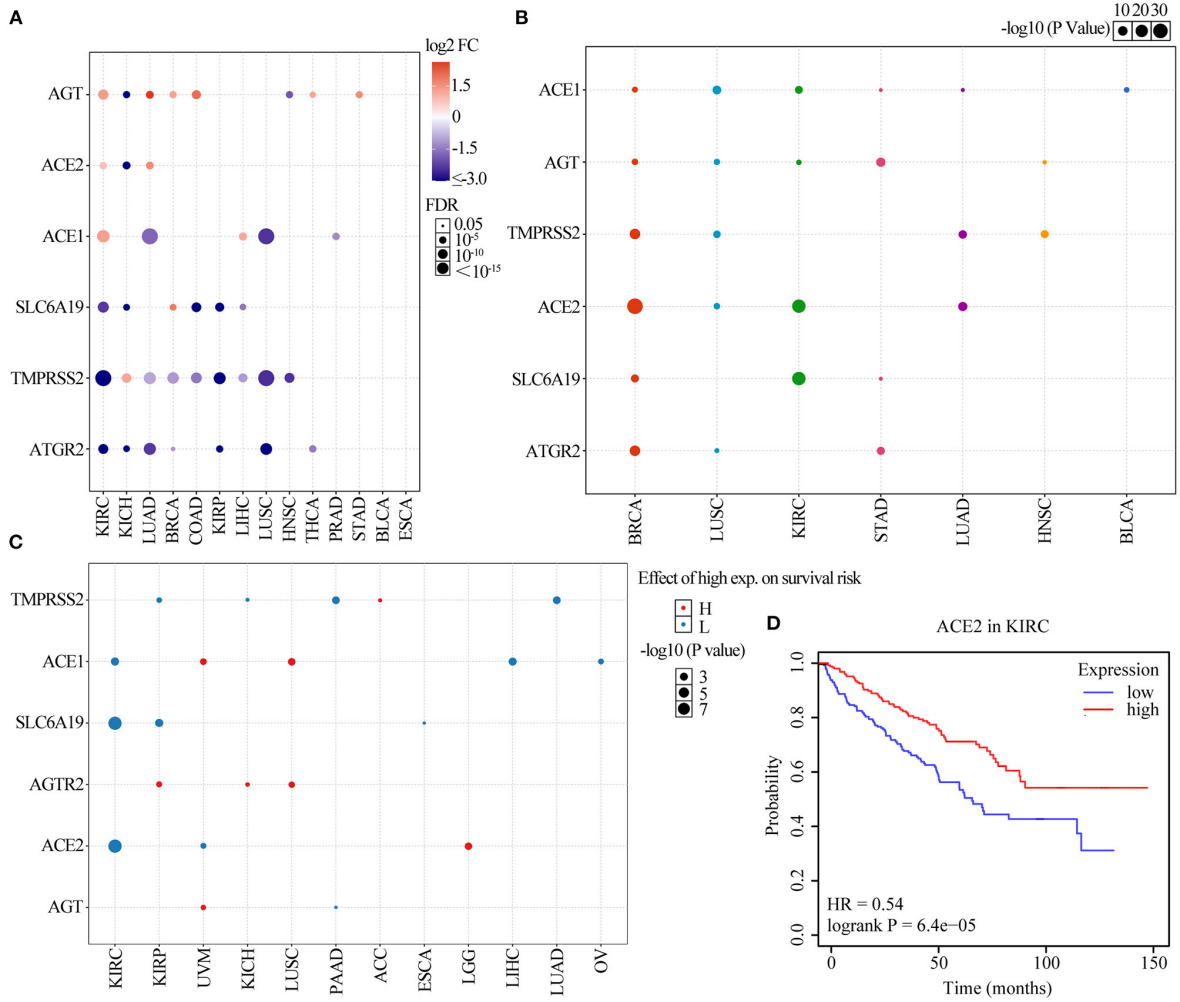
Gene set expression of ACE2 receptor-related regulators. **(A)** Differential expression in 14 solid tumors. The *y*-axis refers to the gene and the *x*-axis refers to the cancer type. The colors range from purple to red, representing the increasing fold change between tumor vs. normal sample, respectively. The size of the dot indicates the degree of significance. **(B)** A graph showing how each subtype is affected by ACE2 receptor-related regulator mRNA expression in seven solid tumors, where each gene can have differential expression in each regulator subtype. **(C)** Survival analysis of ACE2 receptor-related regulators. The dot size represents the significance of a gene affecting survival in each cancer type, and the *p*-value is obtained from a Kaplan–Meier analysis. The red dot color indicated the worse of the high or low expression in the cancer types and the blue dot indicates low expression. **(D)** The survival of ACE2 gene in KIRC.

Six ACE2 receptor-related regulators, namely, TMPRSS2, AGT, ACE1, SLC6A19, ACE2, and AGTR2, were identified and analyzed in this study. We first explored the differential expression of the six receptor-related regulators across cancers based on the TCGA expression data. As shown in Figure 1A, the regulators were identified as having significantly abnormal expression in 14 solid cancers. Expression of TMPRSS2 in KIRC, LUAD, BRCA, COAD, KIRP, LIHC, LUSC, and HNSC; ACE1 in LUAD and LUSC; AGT in KICH and HNSC; ACE2 in KICH; SLC6A19 in KIRC, KICH, COAD, KIRP, and LIHC; and AGTR2 in KIRC, KICH, LUAD, BRCA, KIRP, LUSC, and THCA was significantly downregulated (*p* <0.001). However, expression of AGT in KIRC, LUAD, BRCA, COAD, THCA, and STAD; ACE2 in KIRC and LUAD; ACE1 in KIRC and LIHC; SLC6A19 in BRCA; and TMPRSS2 in KICH was significantly upregulated (*p* <0.001). To further identify the expression of clinically relevant genes that affect cancer subtype, regulator gene expression was explored. The regulator expression subtypes were significantly associated with the tumorigenesis of BRCA, LUSC, KIRC, STAD, LUAD, HNSC, and BLCA (Figure 1B; *p* <0.05). ACE2 in BRCA, ACE1 in LUSC and BLCA, ACE2 and SLC6A19 in KIRC, AGT and AGTR2 in STAD, ACE2 and TMPRSS2 in LUAD, and TMPRSS2 in HNSC were the main regulator subtypes. The results indicated that COVID-19 may be more infectious in BRCA, LUSC, KIRC, STAD, LUAD, HNSC, and BLCA patients than in the normal population.

We further explored the effect of regulator expression on cancer survival and found that high expression of TMPRSS2 in ACC; ACE1 in UVM and LUSC; AGTR2 in KIRP, KICH, and LUSC; ACE2 in LGG; and AGT in UVM were associated with poor survival of cancer patients, while expression of TMPRSS2 in KIRP, KICH, PAAD, and LIHC; ACE1 in KIRC, LIHC, and OV; SLC6A19 in KIRC, KIRP, and ESCA; ACE2 in KIRC and UVM; and AGT in PAAD were associated with good survival in cancer patients (Figure 1C and Supplementary Figure 1; *p* <0.05). As shown in Figure 1D, the low expression of ACE2 was significantly associated with poor survival in KIRC (HR = 0.54; *p* = 6.4e−05). These results indicated that the expression of COVID-19 receptor-related regulators may play an important role in the progression and deterioration of cancer with COVID-19.

### Somatic Mutations of ACE2 Receptor Regulators

**Figure 2:**
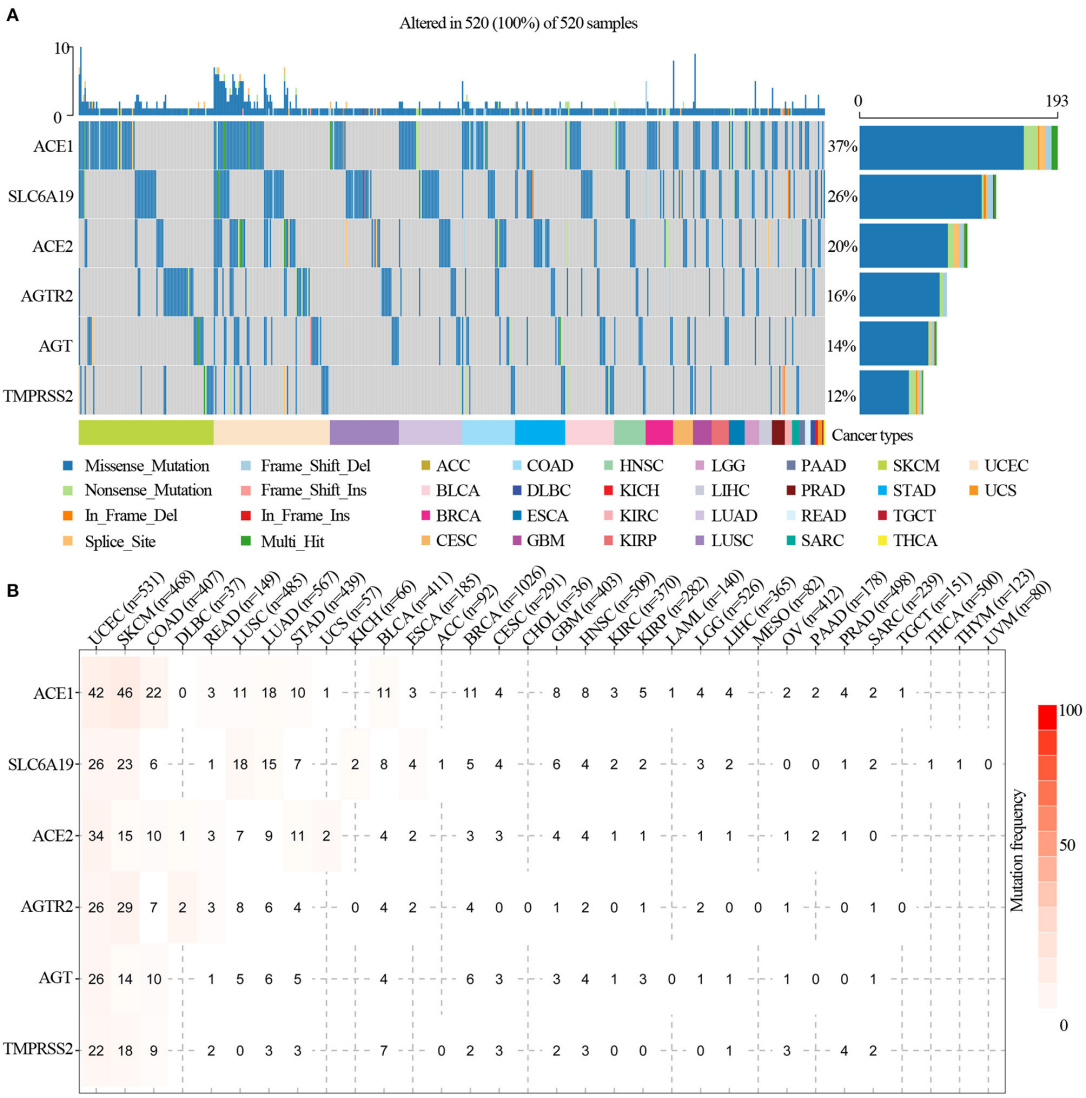
SNV frequency and variant types of ACE2 receptor-related regulators. **(A)** SNV oncoplot. An oncoplot showed the mutation distribution of ACE2 receptor-related regulators and a SNV classification of SNV types (such as missense mutation, frame shift deletion, and non-sense mutation). All selected cancer samples are shown together. Side bar plot and top bar plots show the number of variants in each sample or gene. **(B)** The SNV frequency of genes in cancers. The darker the color, the higher the mutation frequency. Numbers represent the number of samples that have the corresponding mutated gene for a given cancer. “0” indicates that there was no mutation in the gene coding region, and no number indicates that there was no mutation in any region of the gene.

We analyzed ACE2 receptor regulator-related SNP data to detect frequency and variant types in each cancer subtype. As shown in the oncoplot in Figure 2A, the main variant type of the regulators in different cancer subtypes were missense_mutation, in_frame_del, nonsense_mutation, splice_site, in_frame_ins, frame_shift_del, frame_shift_ins, and multi-hit. Regulator SNV frequency was increased in SKCM, UCEC, LUAD, and LUSC. The SNV frequency of the regulators in pan-cancers was 100% (520 out of 520 tumors). The SNV frequency of ACE1, SLC6A19, ACE2, AGTR2, AGT, and TMPRSS2 were 37, 26, 20, 16, 14, and 12%, respectively. SNV percentage analysis indicated that ACE1, SLC6A19, ACE2, AGTR2, AGT, and TMPRSS2 were 42, 26, 34, 26, 26, and 22%, respectively, in UCEC; 46, 23, 15, 29, 14, and 18%, respectively, in SKCM; 11, 18, 7, 8, 5, and 0%, respectively, in LUSC; and 18, 15, 9, 6, 6, and 3%, respectively, in LUAD (Figure 2B). The most frequent mutations were X971_splice/R971W in ACE1, H195Y/X195_splice in ACE2, F430Lfs^*^25 in AGT, R182^*^ in AGTR2, D334N in SLC6A19, and G492S/C in TMPRSS2 (Supplementary Figure 2). In addition, ACE1 mutations found in malignancies were distributed across all exons of ACE1, with several hot spot mutation sites, such as R487H in GBM; R508Q, E510K, and R487C in UCEC; and E510K in UVM (Supplementary Table 1). Pan-cancer mutation prognosis analysis showed that ACE1 and TMPRSS2 mutations were associated with better survival in cancer patients (Supplementary Figure 3; *p* = 0.0273 and 1.18e−10), whereas mutated SLC6A19 was associated poor survival in cancer patients (Supplementary Figure 3; *p* = 1.47e−4). These results indicated that mutations in ACE2 receptor regulators are involved in tumorigenesis and associated with clinical survival.

### CNV of ACE2 Receptor Regulators

**Figure 3:**
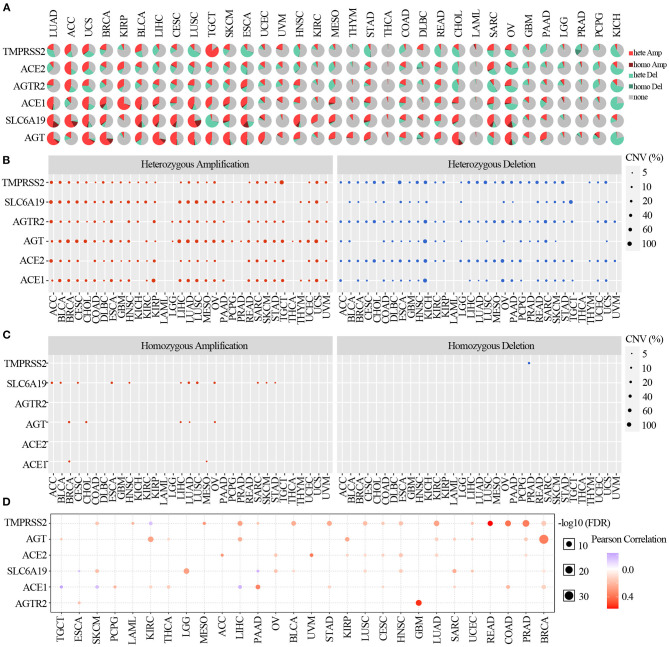
Copy number variation of ACE2 receptor-related regulators. **(A)** CNV pie distribution in 33 cancers. CNV pie plot showed the combined heterozygous/homozygous CNV of each gene in each cancer. A pie represented the proportion of different types of CNV of one gene in one cancer, and different colors represented different types of CNV. Hete Amp, heterozygous amplification; Hete Del, heterozygous deletion; Homo Amp, homozygous amplification; Homo Del, homozygous deletion; None, no CNV. **(B)** Heterozygous CNV profile showing the percentage of heterozygous CNV, including the percentage of amplification and deletion of heterozygous CNV for each gene in each cancer. Only genes with >5% CNV in a given cancer are shown as a point on the figure. **(C)** Homozygous CNV profile showing the percentage of homozygous CNV, including the percentage of amplification and deletion of homozygous CNV for each gene in each cancer. Only genes with >5% CNV in a given cancer are shown as a point on the figure. **(D)** CNV correlation with mRNA. The association between paired mRNA expression and CNV percentage in samples was based on a Pearson product–moment correlation coefficient. The size of the point represents the statistical significance, where the bigger the dot size, the higher the statistical significance.

To identify the CNV change of ACE2 receptor regulators at the chromosome arm level, we analyzed the CNV data of ACE2 receptors from the TCGA database. We found that TMPRSS2, SLC6A19, ATGR2, AGT, ACE2, and ACE1 had >5% CNV amplification or deletion in 33 cancers. As shown in the CNV pie distribution in Figure 3A, TMPRSS2 had 80% heterozygous amplification in TGCT but 63% heterozygous deletion in ESCA; ACE2 had 51% heterozygous amplification in ACC and >50% heterozygous deletion in OV and KICH; and AGT in LUAD, UCS, BRCA, LIHC, CESC, LUSC, SKCM, ESCA, and CHOL; ACE1 in KIRP; and SLC6A19 in ACC and LUSC had almost 50% heterozygous amplification, whereas ACE1 in KICH; SLC6A19 in TGCT; and AGT in KICH had almost 50% heterozygous deletion. To identify the heterozygous/homozygous CNV profile in each cancer, we further analyzed heterozygous/homozygous amplification and heterozygous/homozygous deletion. As shown in Figure 3B, all regulators had heterozygous amplification and deletion. However, homozygous CNV analysis showed that SLC6A19 had homozygous amplification in 12 solid cancers, with TMPRSS2 homozygous deletion only found in PRAD (Figure 3C).

Comparing the relationship between CNV and mRNA expression, the correlation analysis indicated that mRNA expression of each regulator was positively correlated with its CNV in most cancers (*p* <0.05). However, the expression of TMPRSS2 in KIRC; SLC6A19 in ESCA and PAAD; and ACE1 in TGCT, SKCM, and LIHC were negatively correlated with the CNV (*p* <0.05) (Figure 3D). These results indicated that the CNV of ACE2 receptor-related regulators mediated their abnormal expression, which may play an important role in cancer patients with COVID-19.

### Methylation Analysis of ACE2 Receptor Regulators

**Figure 4:**
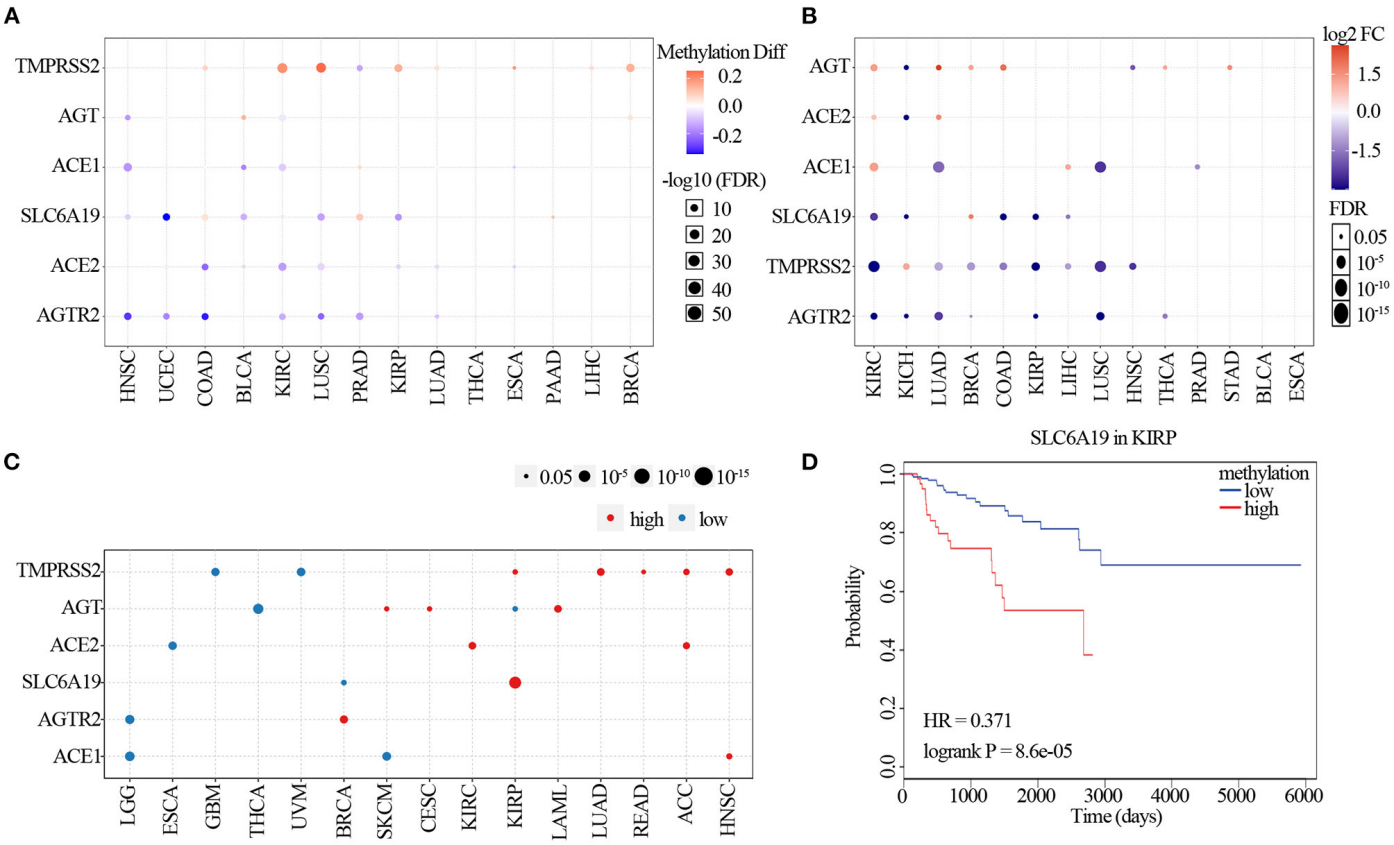
Methylation of ACE2 receptor-related regulators. **(A)** Differential methylation changes in ACE2 receptor-related regulators between tumor and normal samples in each cancer. Blue points represent decreased methylation in tumors and red points represent increased methylation in tumors, where the darker the color, the larger the difference in methylation levels. **(B)** Correlation between methylation and mRNA gene expression. Blue points represent a negative correlation and red points represent a positive correlation, where the darker the color, the higher the correlation. **(C)** Survival difference between samples with ACE2 receptor-related regulators with high and low methylation. Red points represent poorer survival in high methylation groups; blue points were just the opposite. The size of the point represents the statistical significance, where the larger the dot size, the higher the statistical significance. **(D)** Survival analysis of SLC6A19 methylation in KIRP.

We explored the methylation analysis of ACE2 receptor regulators to identify the corresponding epigenetic methylation levels. As shown in Figure 4A, ACE2 in COAD, BLCA, KIRC, LUSC, KIRP, LUAD, and ESCA; AGTR2 in HNSC, UCEC, COAD, KIRC, LUSC, PRAD, and LUAD; SLC6A19 in HNSC; UCEC, BLCA, KIRC, LUSC, and KIRP; ACE1 in HNSC, BLCA, KIRC, and ESCA; AGT in HNSC and KIRC; and TMPRSS2 in PRAD were hypomethylated (*p* <0.05); TMPRSS2 in COAD, KIRC, LUSC, KIRP, LUAD, ESCA, LIHC, and BRCA; AGT in BLCA, LUAD, and BRCA; ACE1 in PRAD; and SLC6A19 in COAD, PRAD, and PAAD were significantly hypermethylated (*p* <0.05). We assessed regulator methylation and mRNA expression through correlation analysis and found that the mRNA expression of AGT in KICH and HNSC; ACE2 in KICH; ACE1 in LUAD, LUSC, and PRAD; SLC6A19 in KIRC, KICH, COAD, KIRP, and LIHC; TMPRSS2 in KIRC, LUAD, BRCA, COAD, KIRP, LIHC, LUSC, and HNSC; and AGTR2 in KIRC, KICH, LUAD, BRCA, KIRP, LUSC, and THCA were negatively correlated with their methylation (*p* <0.05; Figure 4B and Supplementary Figure 4). The mRNA expression of ACE1 in KIRC and LIHC; AGT in KIRC, LUAD, BRCA, COAD, THCA, and STAD; ACE2 in KIRC and LUAD; AGT in KIRC, LUAD, BRCA, COAD, THCA, and STAD; SLC6A19 in BRCA; and TMPRSS2 in KICH were positively correlated with their methylation (*p* <0.05; Figure 4B and Supplementary Figure 4). Prognosis analysis showed that hypermethylation of AGTR2 in BRCA; AGT in SKCM, CESC, and LAML; TMPRSS2 in KIRP, LUAD, READ, ACC, and HNSC; SLC6A19 in KIRP; ACE2 in ACC; and ACE1 in HNSC were associated with poor survival. Hypermethylation of TMPSS2 in GBM and UVM; AGT in THCA and KIRP; ACE2 in ESCA; SLC6A19 in BRCA; AGTR2 in LGG; and ACE1 in LGG and SKCM were associated with good survival (*p* <0.05; Figure 4C). As shown in Figure 4D, the hypermethylation of SLC6A19 was significantly associated with poor survival in KIRP (*p* = 8.6e−05; Figure 4D).

### Pathway Activity Analysis

**Figure 5:**
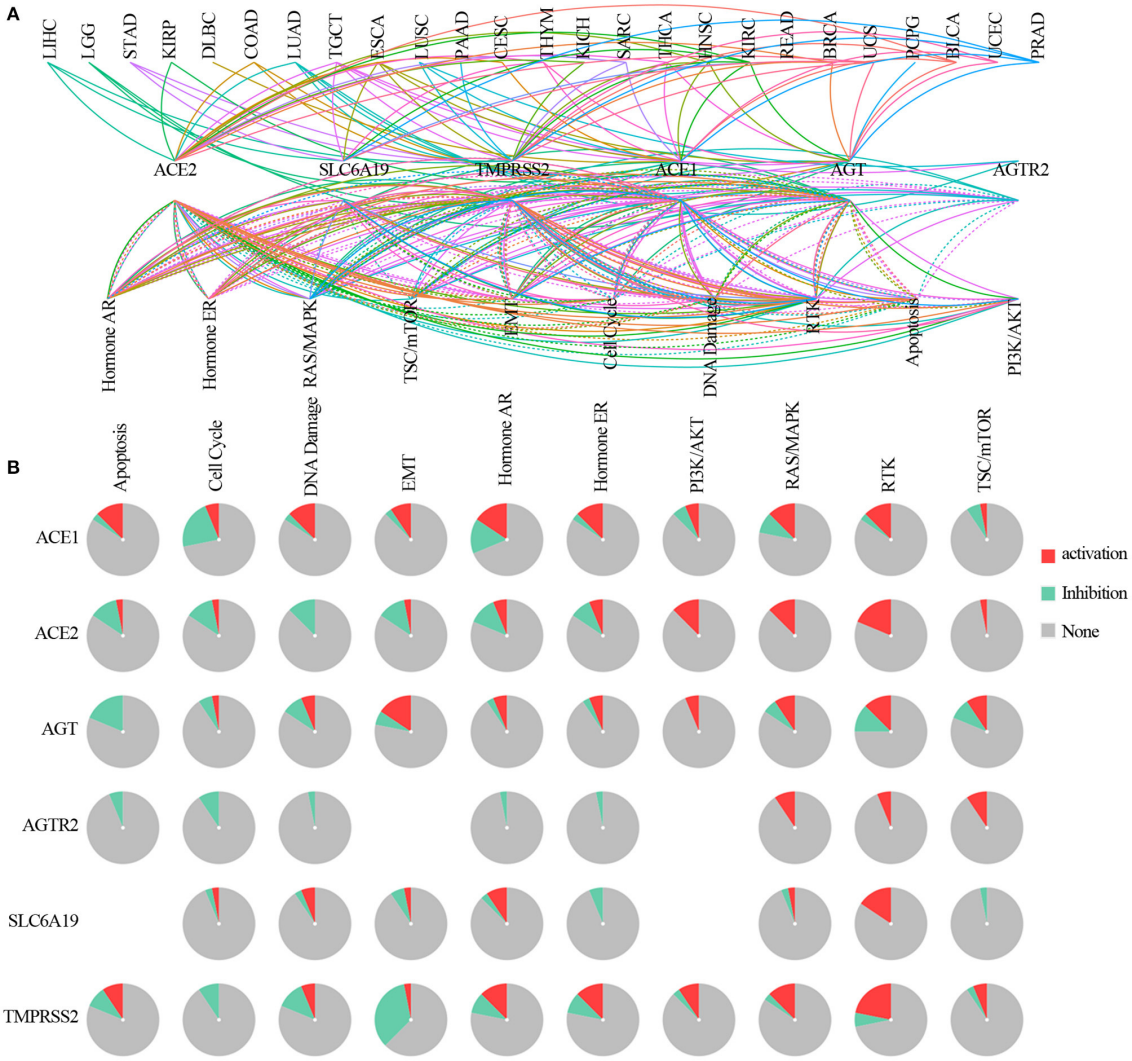
The pathway network between ACE2 receptor-related regulators. **(A)** A line represents a connection between different pathways, where a solid line represents activation and a dashed line represents inhibition. Color of line represented different cancer types. **(B)** Global percentage of cancers in which ACE2 receptor-related regulators had an effect on the pathway among 32 cancer types, obtained as follows: number of activation or inhibition cancer types/32 ×100%.

The pathway relation network indicated that ACE2 receptor-related regulators were involved in TSC/mTOR, RTK, RAS/MAPK, PI3K/AKT, hormone ER, hormone AR, EMT, DNA damage response, cell cycle, and apoptosis pathways (Figure 5A). The global percentage of cancers in which regulators have an effect on a pathway showed that ACE1 was involved in the activation of apoptosis, DNA damage, epithelial–mesenchymal transition (EMT), hormone ER, hormone AR, RAS/MAPK, and RTK pathways and the inactivation of the cell cycle and TSC/mTOR pathways. ACE2 was associated with the activation of PI3K/AKT, RAS/MAPK, and TSC/mTOR pathways, and with the inactivation of the cell cycle, DNA damage, EMT, and hormone AR pathways. AGT was associated with the activation of the EMT pathway and the inactivation of apoptosis. AGTR2 was associated with the activation of RAS/MAPK, RTK, and TSC/mTOR pathways and the inactivation of apoptotic, cell cycle, DNA damage, hormone ER, and hormone AR pathways. SLC6A19 was involved in the activation of RTK and hormone AR pathways and the inactivation of hormone ER and TSC/mTOR pathways. TMPRSS2 was associated with the activation of the RTK pathway and inactivation of EMT (Figure 5B). As ACE2 receptor-related regulators are often mutated in UCEC, we further analyzed the global percentage of pathway activity in UCEC. We found that ACE1 was mostly involved in the inhibition of the cell cycle (21% inhibition vs. 7% activation) and activation of RAS/MAPK (9% inhibition vs. 13% activation). ACE2 was mainly involved in the inhibition of hormone AR (12% inhibition vs. 7% activation) and activation of the RTK pathway (0% inhibition vs. 19% activation). AGT was associated with inhibition of apoptosis (18% inhibition vs. 0% activation) and activation of EMT (6% inhibition vs. 16% activation). TMPRSS2 was mainly involved in the inhibition of the DNA damage response (12% inhibition vs. 7% activation) and EMT (34% inhibition vs. 4% activation), while it was associated with the activation of hormone AR (9% inhibition vs. 13% activation), hormone ER (9% inhibition vs. 13% activation), and RTK (6% inhibition vs. 22% activation) pathways (Supplementary Figure 5). These results indicated that the abnormal expression of ACE2 receptor-related regulators mediated the abnormal activation of cancer-related signaling pathway, which played different roles in regulating tumorigenesis and progression.

### miRNA Regulation Analysis

**Figure 6:**
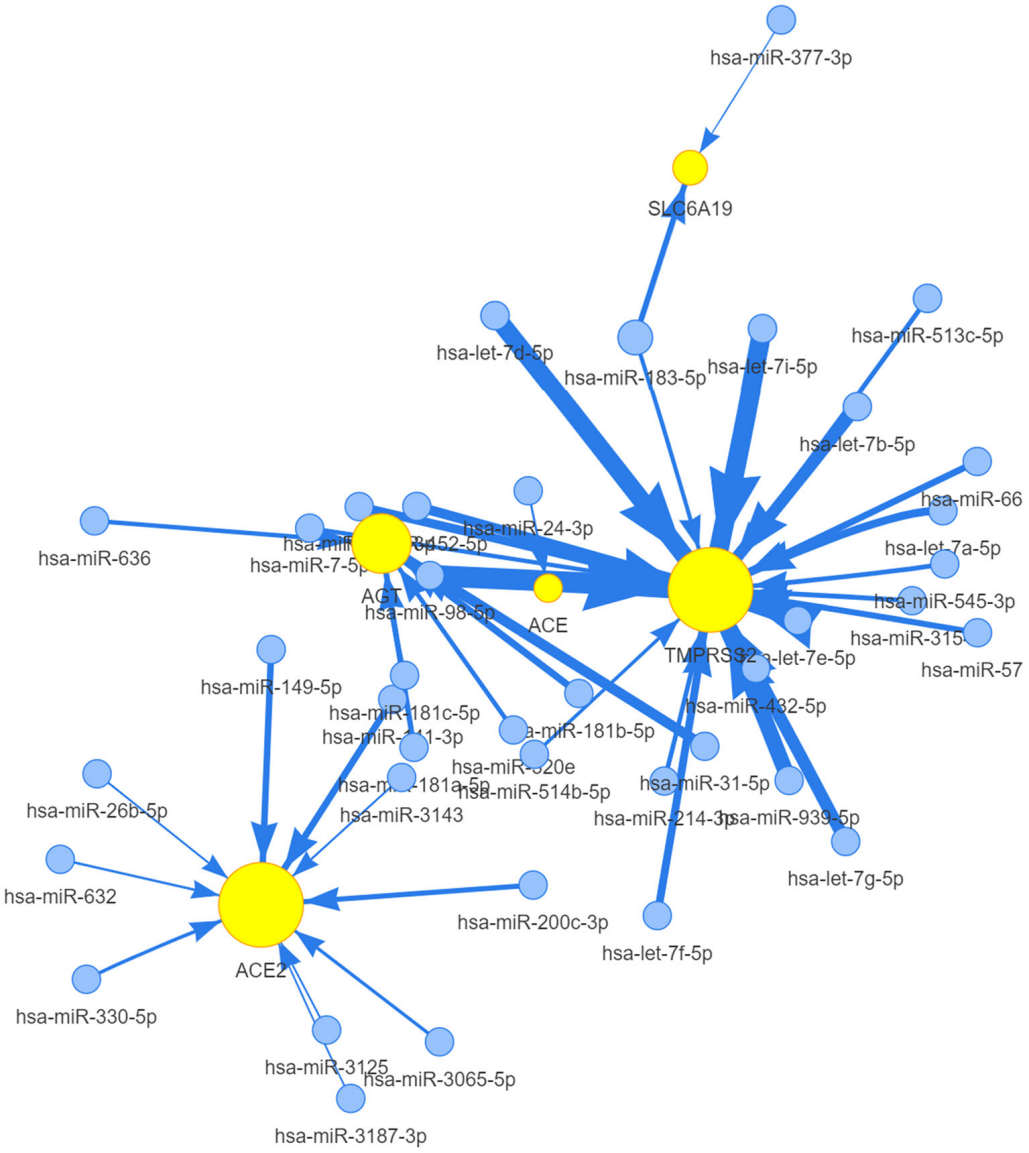
The miRNA network of ACE2 receptor-related regulators. A connection node between miRNA and one regulator represents miRNA regulation of a gene. Node size is positively correlated to the node's degree similar to networkD3, and edge width is defined by the absolute value of the correlation coefficient.

To clarify any miRNA regulation of ACE2 receptor-related regulators, visNetwork was used to generate miRNA regulation networks. As shown in Figure 6, hsa-miR-98-5P, hsa-let-7a-5P, hsa-miR-665, hsa-miR-432-5P, hsa-let-7b-5P, hsa-let-7d-5p, hsa-let-7g-5p, hsa-miR-545-3P, hsa-miR-452-5P, hsa-miR-939-5P, hsa-miR-7-5P, hsa-miR-513c-5P, hsa-miR-514-5P, hsa-miR-664a-3P, and hsa-let-7i-5p, hsa-let-7f-5P, hsa-let-7e-5P, hsa-miR-214-3P, hsa-miR-3154, hsa-miR-573, and hsa-miR-183-5P were negatively correlated with TMPRSS2 expression (*p* <0.05); hsa-miR-31-5p, hsa-miR-181a-5p, hsa-miR-181b-5p, hsa-miR-181C-5p, hsa-miR-636, and hsa-miR-320e were negatively correlated with AGT; and hsa-miR-632, hsa-miR-330-5p, hsa-miR-200c-3p, hsa-miR-141-3p, hsa-miR-632, hsa-miR-26b-5p, hsa-miR-149-5p, hsa-miR-3125, hsa-miR-3143, hsa-miR-3187-3p, hsa-miR-200c-3p, and hsa-miR-3065-5p were negatively regulated with the expression of ACE2 (*p* <0.05); hsa-miR-183-5p and hsa-miR-377-3p were negatively regulated with the expression of SLC6A19 (*p* <0.05); and hsa-miR-24-3p were negatively regulated with the expression of ACE1 (*p* <0.05). These results indicated that the miRNA regulation network mediated ACE2 receptor-related regulators, which may be involved in the progression of cancer in patients with COVID-19.

### Drug Sensitivity Analysis

**Figure 7:**
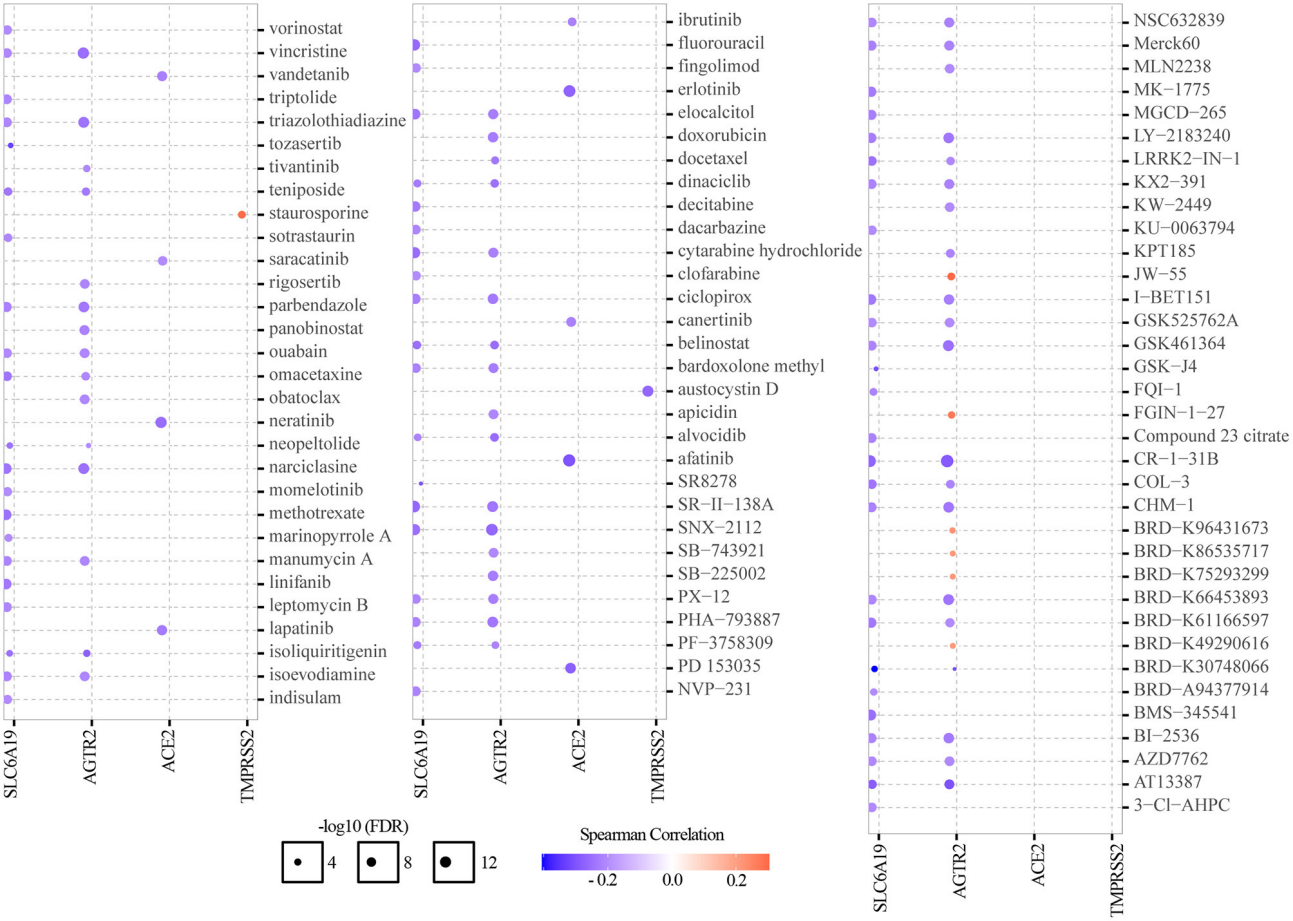
Drug sensitivity analysis of ACE2 receptor-related regulators. The gene set drug resistance analysis from CTRP IC_50_ drug data. Spearman's correlation represented how the gene expression correlates with a drug. A positive correlation means that a gene with high expression was resistant to a drug, and *vice versa*.

Genomic aberrations influenced clinical response to treatment and are potential biomarkers for drug screening in cancer. To know the role of ACE2 receptor-related regulators on chemotherapy or targeted therapy, drug sensitivity and gene expression profiling data of cancer cell lines from the CTRP were integrated. Spearman's correlation analysis showed that drug sensitivity toward vincristine, teniposide, ouabain, docetaxel, doxorubicin, erlotinib, afatinib, AZD7762, and AT13387 correlated with the expression of AGTR2, SLC6A19, ACE2, and TMPRSS2 (negative correlation with IC_50_). Drug resistance toward staurosporine correlated with the expression of TMPRSS2, JW55, FGIN-1-27, BRD-K96431673, BRD-K86535717, BRD-K75293299, and BRD-K49290616 (positive correlation with IC_50_) (Figure 7). These results indicated that the abnormal expression of ACE2 receptor-related regulators may mediate sensitivity to chemotherapy and targeted drug therapy.

## Discussion

COVID-19 is a global health emergency problem with a large number of confirmed cases and deaths that are much greater than any infection in recent decades. Condition severity and mortality have been identified as being significantly higher in patients with other comorbidities, such as cancer (30–32). As care for chronic conditions, such as cancer, still needs to continue during the pandemic, it is necessary for healthcare providers to determine which type of cancer will put patients at a higher risk of exhibiting severe forms of the COVID-19 infection. Patients have also had to balance the risks and benefits of cancer-directed interventions within the context of the added risk of contracting COVID-19. In this study, we comprehensively analyzed the genomic and prognostic characteristics of six COVID-19 receptor-related regulators, where we found that genetic and epigenetic alterations, and an miRNA network of COVID-19 receptor-related regulators, led to their abnormal expression, which correlated significantly with the activation of cancer hallmark-related pathways and clinical survival. Targeting these COVID-19 receptor-related regulators may be an important method to treat cancer patients with COVID-19.

We firstly explored the genetic alterations and prognoses of these regulators and found that the abnormal expression was associated with clinical prognosis. Our results indicated that there were 14 tumor types that differentially expressed one or more of these regulators. The regulators were highly expressed in normal mucosal epithelial tissue, such as kidney, urinary bladder, and mucocutaneous and gastrointestinal tract, which was consistent with ACE2 protein distribution. This co-expression pattern further validated that the SARS-CoV-2 entry process requires the interaction of these regulators. By tracking the genetic differences in these six regulators, we found that missense mutations were the main mutation type in SNV, with ACE1 having the highest mutation frequency in cancer. In addition, ACE1 mutations in malignancies were distributed across all exons of ACE1 with several hot spot mutation sites. ACE mutations have been reported to be involved in a number of lymph node metastases of gastric cancer (33) and associated with a worse prognosis in prostate cancer (34). However, there was also non-conformity between genomics alternation and clinical prognosis. Thus, we speculated that genetic and epigenetic alteration of the regulators may cause gene dysfunction and promote tumorigenesis in certain contexts.

Further investigation into the biological function of the regulators identified several pathways, including TSC/mTOR, RTK, RAS/MAPK, PI3K/AKT, hormone ER, hormone AR, EMT, DNA damage response, cell cycle, and apoptosis pathways, that were significantly enriched in cancers. In UCEC, different ACE2 receptor-related regulators were associated with different cancer-related signaling pathways. For example, TMPRSS2 was involved in the activation of the RTK pathway and AGTR2 was associated with the inhibition of the cell cycle and the apoptosis pathway. Recent studies identified that Ang II can also promote cell growth and proliferation *via* the transforming growth factor-beta (35), RTK (36), and mTOR pathways (37). Activation of Ang II receptor in cancer cell lines resulted in increased MAPK activation, JAK-STAT signaling, and cell proliferation (38, 39). Thus, activation and inhibition of cancer-related signaling pathways mediated by ACE2 receptor-related regulator molecular networks played different roles in tumorigenesis and prognosis.

In clinical applications, dexamethasone, which can reduce inflammation, and remdesivir, which can inhibit viral replication, have been widely used to decrease the mortality in cancer patients with COVID-19 (40, 41). There are currently no effective drugs for COVID-19. There is an urgent need for therapeutic interventions, especially for cancer patients with weakened immune systems. Our drug sensitivity analysis identified that ACE2 receptor-related regulator expression levels were also involved in drug sensitivity. Vorinostat is an anticancer histone deacetylase (HDAC) inhibitor and has previously been shown to have anti-fibrotic effects and can reduce the risk of acute respiratory deterioration by upregulating ACE2 expression (42, 43). Erlotinib, an epidermal growth factor receptor (EGFR) inhibitor, has been reported to inhibit endocytosis and intracellular trafficking of multiple viruses, including hepatitis C, dengue, and Ebola, exerting broad-spectrum antiviral effects by increasing ACE2 expression (44). Thus, we speculate that targeting ACE2 receptor-related regulators will become an ideal approach in cancer treatment. However, variations of ACE2 receptor-related regulators exist at all regulation levels, including genetics and epigenetic alterations, mRNA expression, miRNA networks, and pathway correlations. These variations may alter drug effects, treatment responses, and patient survival. Thus, the potential mechanisms of each drug's effect on ACE2 receptor-related regulator expression and cancer progression require further investigation.

## Conclusion

In conclusion, our findings indicate the need for precautions for and protection of cancer patients during the COVID-19 pandemic. However, the balance between the risks and benefits of cancer-directed interventions should be reassessed. Thus, targeting ACE2 receptor-related regulators could be a promising strategy against cancer patients with COVID-19.

## Data Availability Statement

The original contributions presented in the study are included in the article/Supplementary Material, further inquiries can be directed to the corresponding author/s.

## Author Contributions

JZ, HJ, and KD performed all experiments, prepared the figures, and drafted the manuscript. JZ, TX, BW, and BC participated in the data analysis and results interpretation. JZ, KD, CC, YY, and JY designed the study and participated in the data analysis. All authors contributed to the article and approved the submitted version.

## Conflict of Interest

The authors declare that the research was conducted in the absence of any commercial or financial relationships that could be construed as a potential conflict of interest.

## Publisher's Note

All claims expressed in this article are solely those of the authors and do not necessarily represent those of their affiliated organizations, or those of the publisher, the editors and the reviewers. Any product that may be evaluated in this article, or claim that may be made by its manufacturer, is not guaranteed or endorsed by the publisher.

## Supplementary Material

Supplementary Figure 1 Overall survival of ACE2 receptor related regulators in cancers.

Supplementary Figure 2 Mutational map of ACE2 receptor related regulators in cancers.

Supplementary Figure 3 Overall survival of the mutations of ACE2 receptor related regulators in cancers.

Supplementary Figure 4 Correlation analysis of methylation and mRNA expression of ACE2 receptor related regulators.

Supplementary Table 1 Mutations of ACE2 receptor related regulators in cancers.
