# Supplementary figures and images for: Pan-Cancer Analysis of Genomic and Prognostic Characteristics Associated With Coronavirus Disease 2019 Regulators
Source: Front Med (Lausanne). 2021 Aug 11;8:662460. doi: 10.3389/fmed.2021.662460 (PMC8385656; doi:10.3389/fmed.2021.662460)

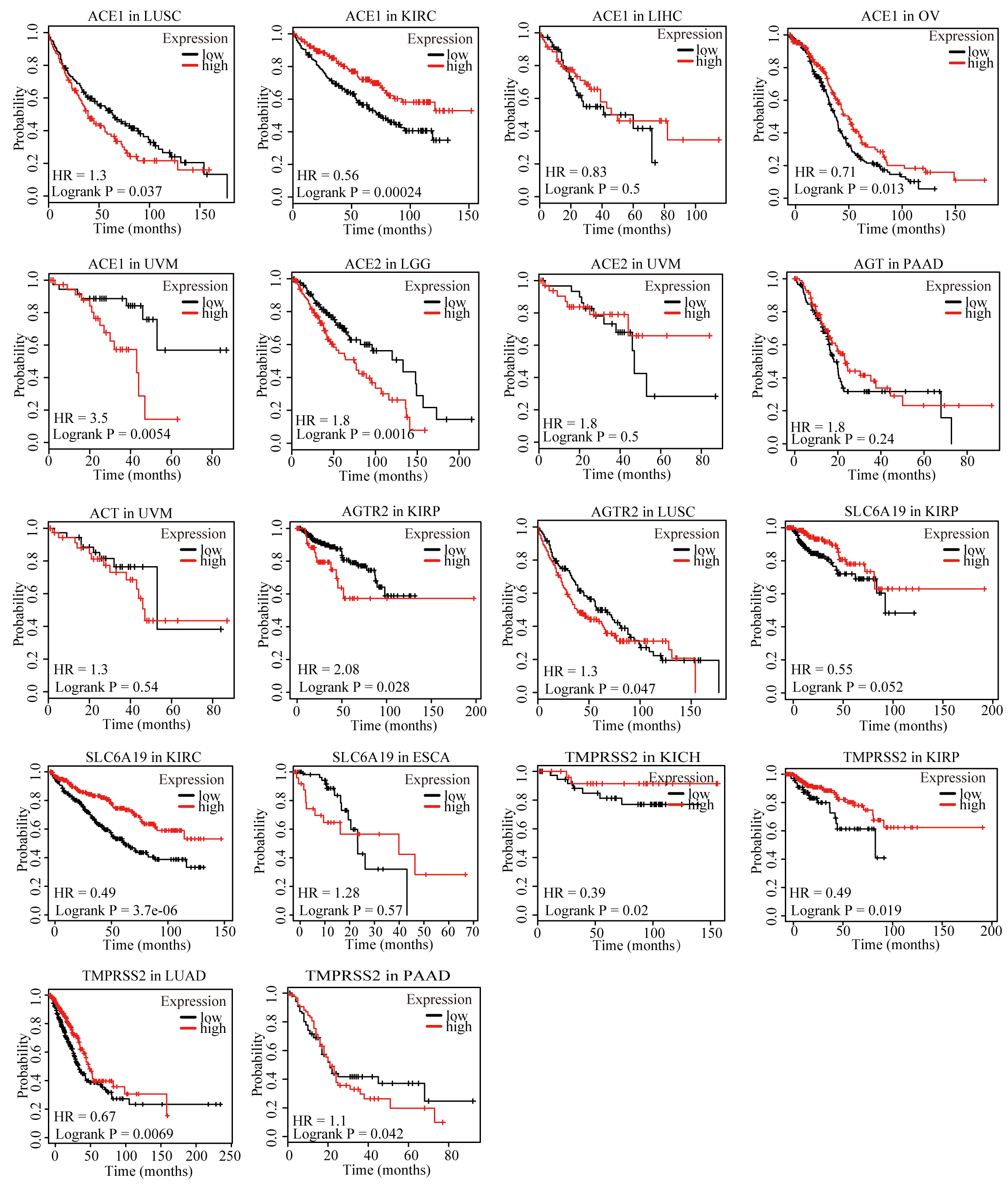

Supplement: Supplementary Figure 1 — Overall survival of ACE2 receptor related regulators in cancers. [file Image_1.TIF]

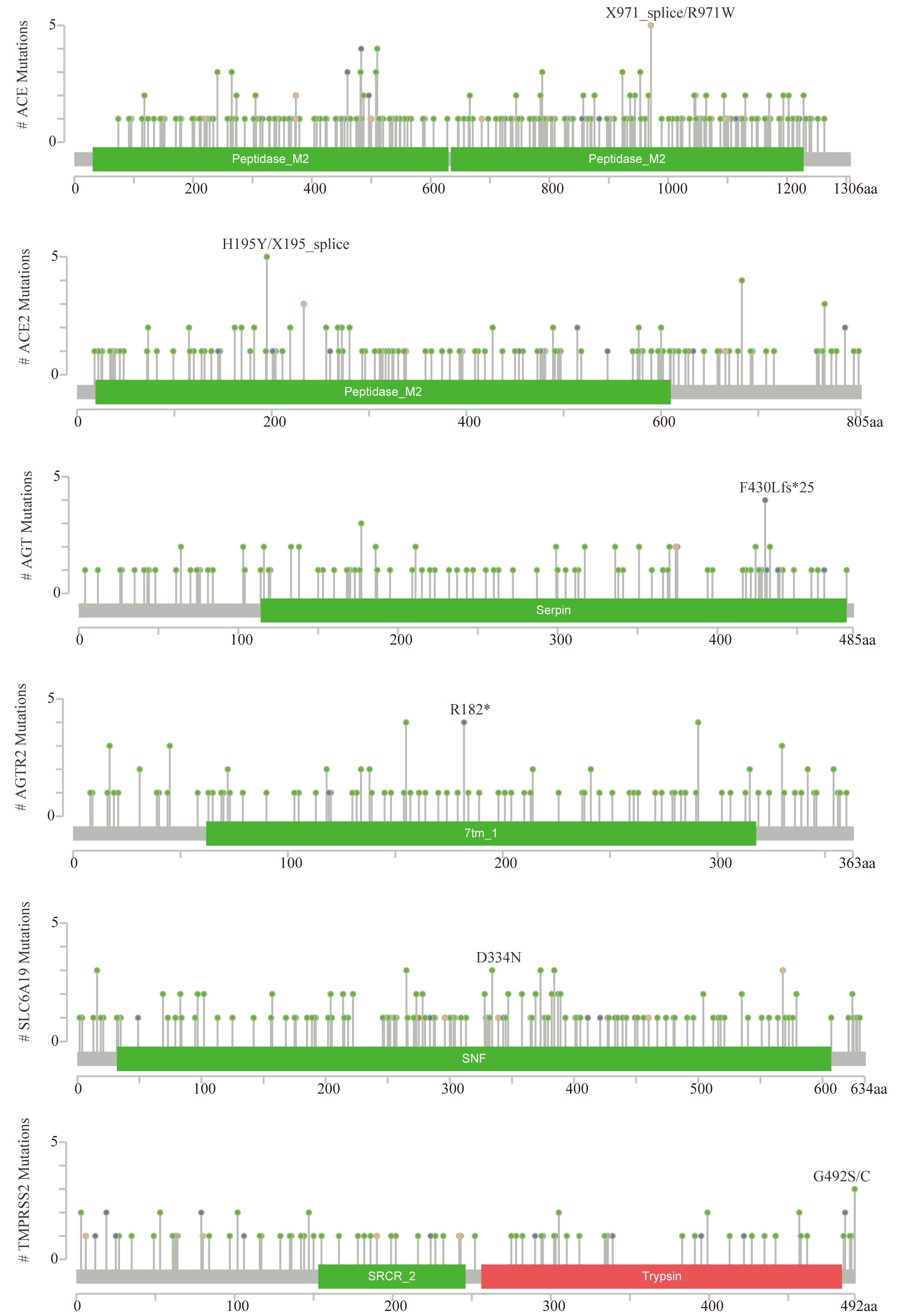

Supplement: Supplementary Figure 2 — Mutational map of ACE2 receptor related regulators in cancers. [file Image_2.TIF]

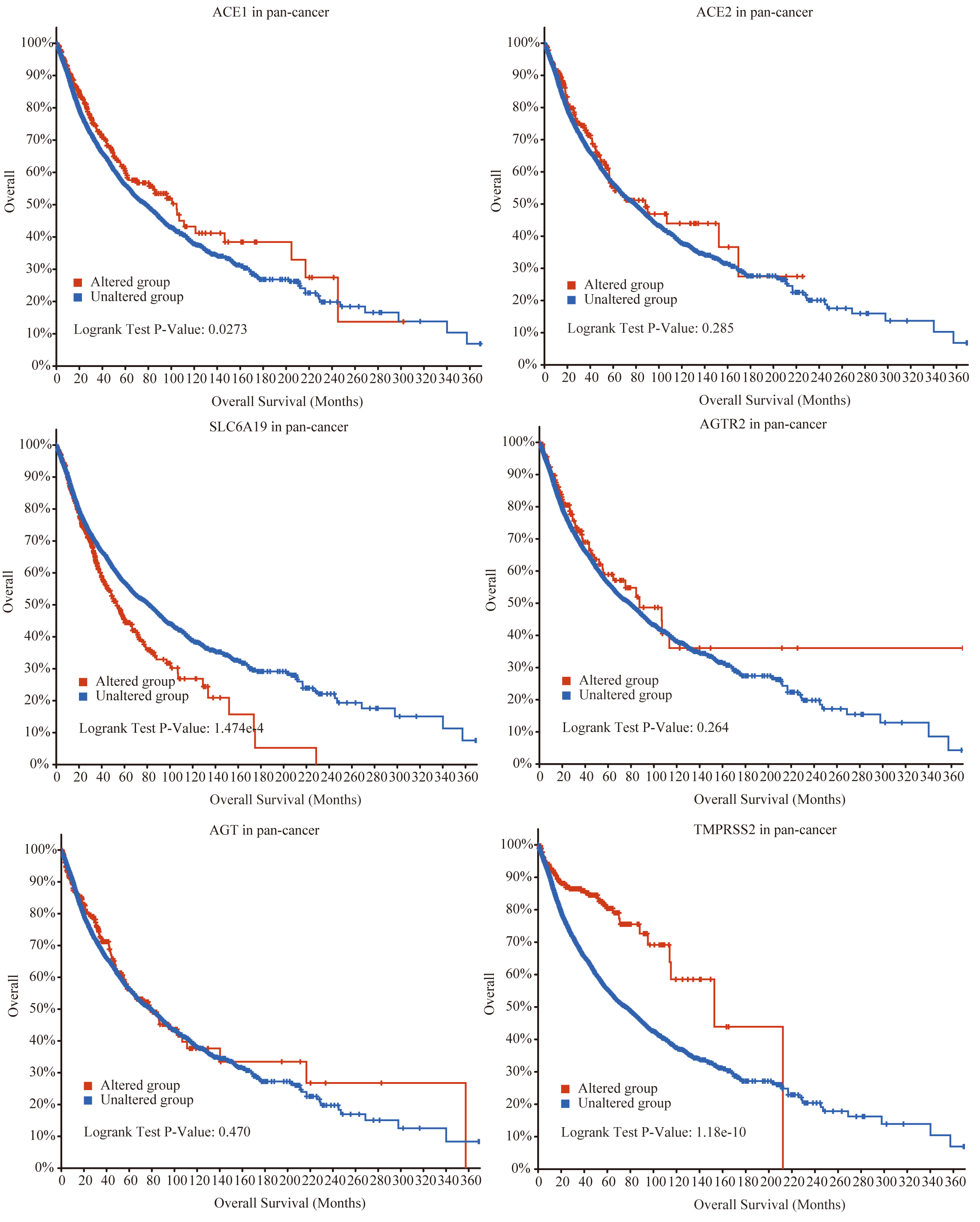

Supplement: Supplementary Figure 3 — Overall survival of the mutations of ACE2 receptor related regulators in cancers. [file Image_3.TIF]

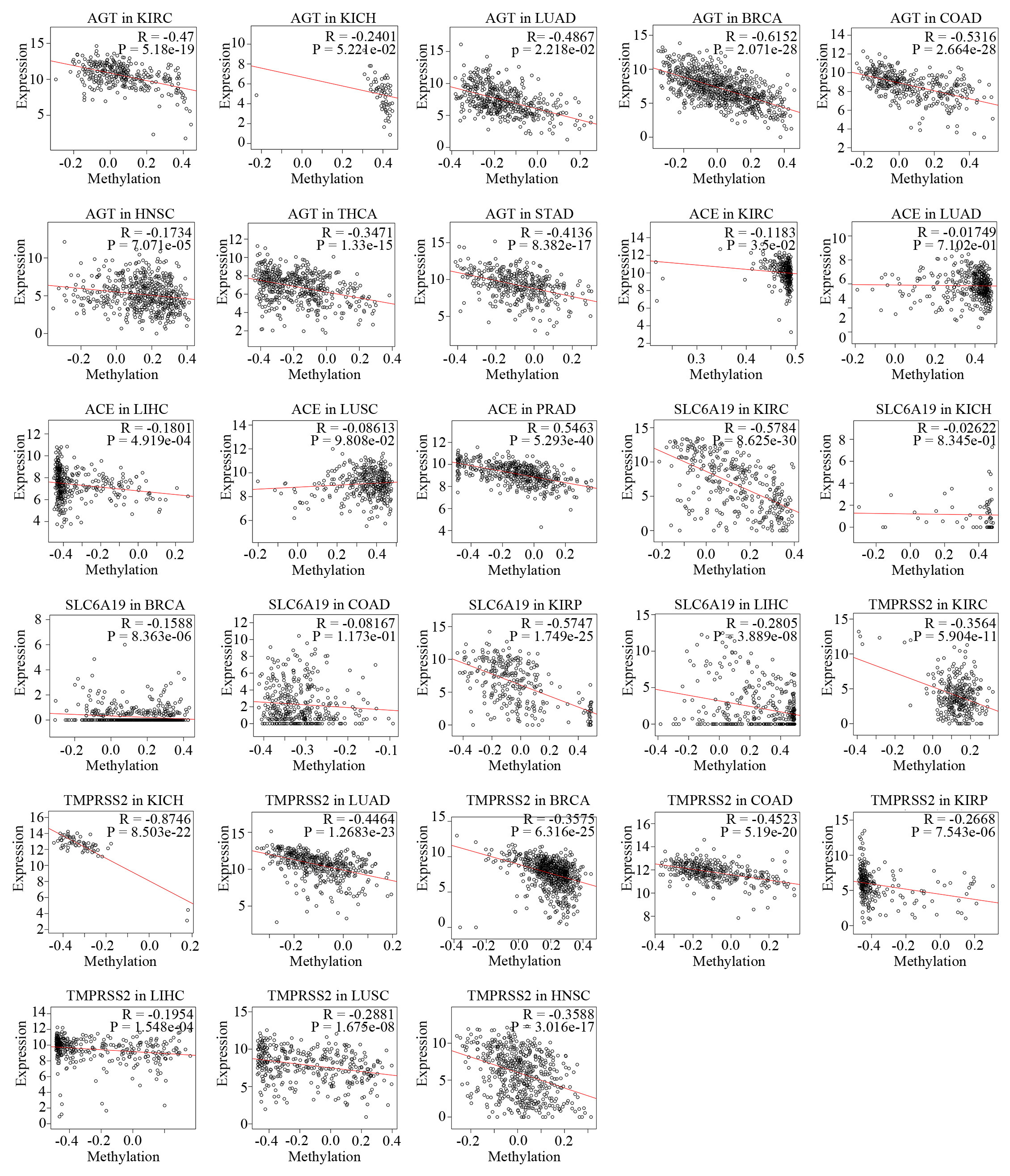

Supplement: Supplementary Figure 4 — Correlation analysis of methylation and mRNA expression of ACE2 receptor related regulators. [file Image_4.TIF]

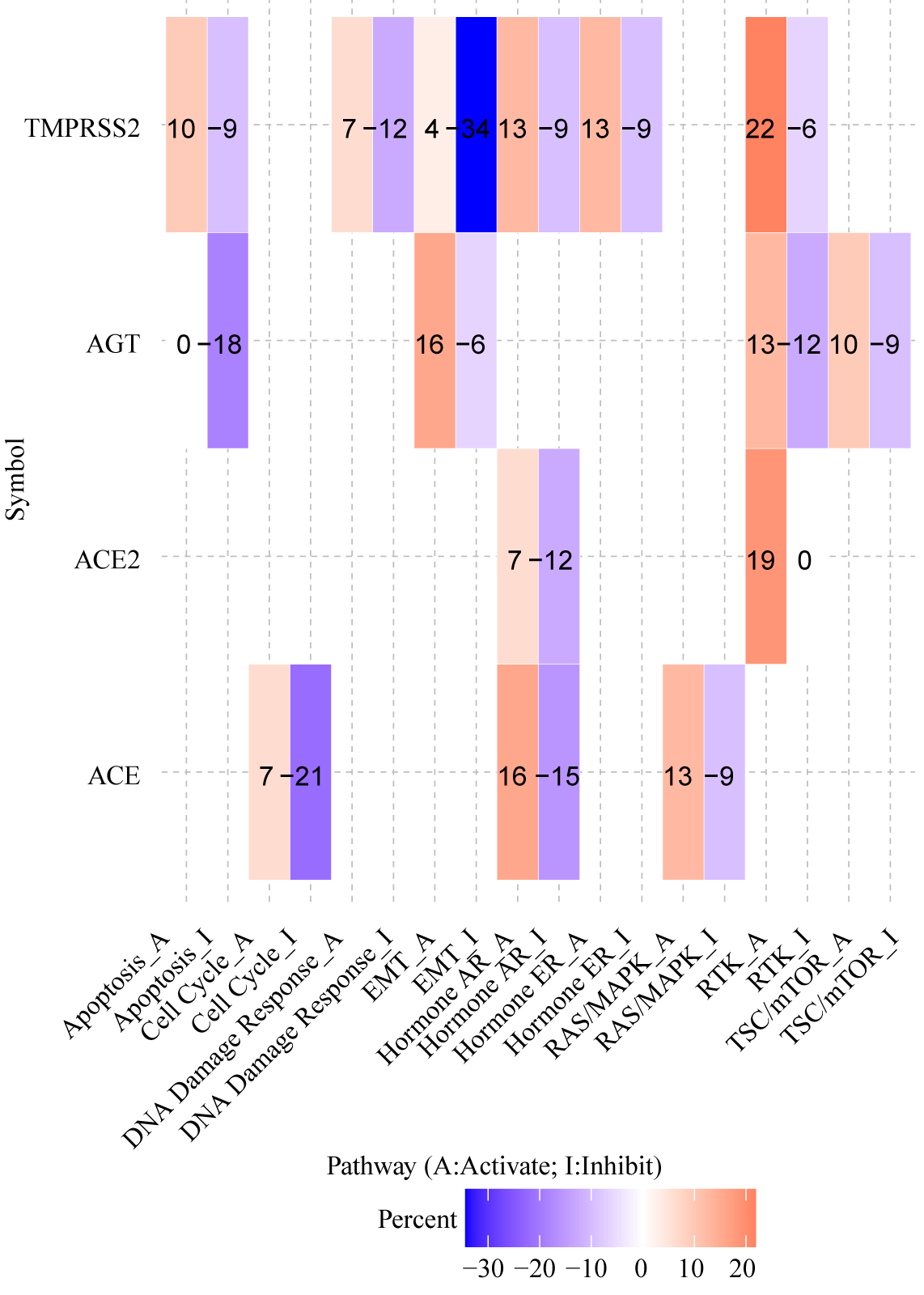

Supplement: Supplementary file 6 [file Image_5.TIF]
